# One in One Million—A Case of Pleural Disease

**DOI:** 10.1093/icvts/ivaf286

**Published:** 2026-02-02

**Authors:** Tara Byrne, Silvie Blaskova, Alan Soo

**Affiliations:** Cardiothoracic Department, Galway University Hospital, Galway, Ireland; Medical Oncology Department, Galway University Hospital, Galway, Ireland; Cardiothoracic Department, Galway University Hospital, Galway, Ireland; Medical Oncology Department, Galway University Hospital, Galway, Ireland

**Keywords:** pleural disease, vascular tumour, epithelioid hemangioendothelioma

## Abstract

This case report presents an instance of pleural epithelioid hemangioendothelioma (EHE), a vascular tumour with an incidence of less than 1% among vascular tumors. The patient, a 43-year-old man, presented with a right-sided pleural effusion, longstanding neck and shoulder pain, and worsening pleuritic chest pain. Initial imaging revealed a left infra-clavicular soft tissue mass, pleural thickening, and pulmonary nodules suggestive of metastases. Despite inconclusive initial biopsies, immunohistochemistry and an international pathology review confirmed EHE, characterized by CAMTA1 expression and WWTR1 CAMTA1 fusion. The pleural involvement indicated metastatic disease, leading to a poor prognosis. Treatment with the MEK inhibitor trametinib was initiated, but the patient died within 3 months. This case underscores the diagnostic challenges of EHE due to its rarity and variable clinical presentation, which often delays diagnosis until advanced stages. The report highlights the aggressive nature of pleural EHE and the lack of standardized treatments, emphasizing the need for early recognition.

## INTRODUCTION

Epithelioid hemangioendothelioma (EHE) is a rare vascular tumour that was first described in 1975 by Dail and Liebo.^1^ It was frequently referred to as an aggressive bronchoalveolar cell carcinoma. Today, EHE continues to be a challenging disease for specialists to diagnose and is a rare sighting in pleural diseases. Epithelioid hemangioendothelioma is primarily a rare vascular tumour described in appearance as epithelioid and histiocytic, originating from vascular endothelial or pre-endothelial cells. It represents 1% of all vascular tumours.^1^ Epithelioid hemangioendothelioma can present itself in multiple places in the body, such as the liver, lungs, skin, bone, spleen, lymph nodes, and the pleura.

## CASE DISCUSSION

This case presents a 43-year-old man who presented with a right-sided pleural effusion of unknown cause. Further enquiry revealed a longstanding history of neck pain, left-sided shoulder pain, worsening complaints of pleuritic chest pain, and shortness of breath, resulting in a GP referral to the medical assessment unit. Interestingly, this man had previously complained of severe neck pain approximately ten years prior. Unfortunately, despite extensive investigations, there was no definitive diagnosis.

There is a background history of Addison’s disease. Socially, he was a non-smoker, independent, living with family, with no concerning occupational or family history.

## CLINICAL FINDINGS- DIAGNOSTIC ASSESSMENT

Initial chest x-ray indicated a moderate-sized right-sided pleural effusion with associated right basal atelectasis. With no clinical Indication for the cause of the pleural effusion, a CT thorax was warranted for consideration of a malignant process.

The CT Thorax reported to note an Ill-defined soft tissue mass in the left infraclavicular region. Enlarged right internal mammary lymph node, Ill-defined right supra-diaphragmatic nodes measuring up to 1.2 × 1.0 cm, and a prominent right hilar node measuring up to 8 mm. Moderate-sized right pleural effusion with associated compressive atelectasis in the right lower lobe and mild left basal atelectasis was evident. There were multiple bilateral pulmonary nodules, suspicious for metastases, with obvious atrophy of the left pectoralis muscles. Finally, multiple venous collaterals in the upper left chest wall were noted. Aspiration of the pleural fluid was negative for malignancy.

A CT Neck concluded that there was an Ill-defined left infra-clavicular soft tissue mass encasing and occluding the left subclavian vein, with erosion of the adjacent clavicle and associated atrophy of the left pectoralis muscles. A previous MRI, captured two months prior, of the brachial plexus showed evidence of a benign haemangioma in the C5 posterior superior hemi-vertebra, but this was not suspected as the cause of symptoms. This was not evident on an MRI the year prior.

This case was discussed at the local lung cancer MDM when the recommendation was for a Thoracic surgery review for possible VATS and surgical biopsy. For completeness, a surgical pleural biopsy via VATS and a biopsy of the lesion in the left infra-clavicular region were undertaken. The sample was compared with a wide panel of immunohistochemistry, which effectively ruled out the possibility of carcinoma or melanoma in the material, and there was no morphological evidence of lymphoma. The possibility of a sarcomatous lesion did remain within the differential, and this included lesions such as desmoid fibromatosis and synovial sarcoma. Other differential diagnoses that were considered included mesothelioma.

**Figure 1.**
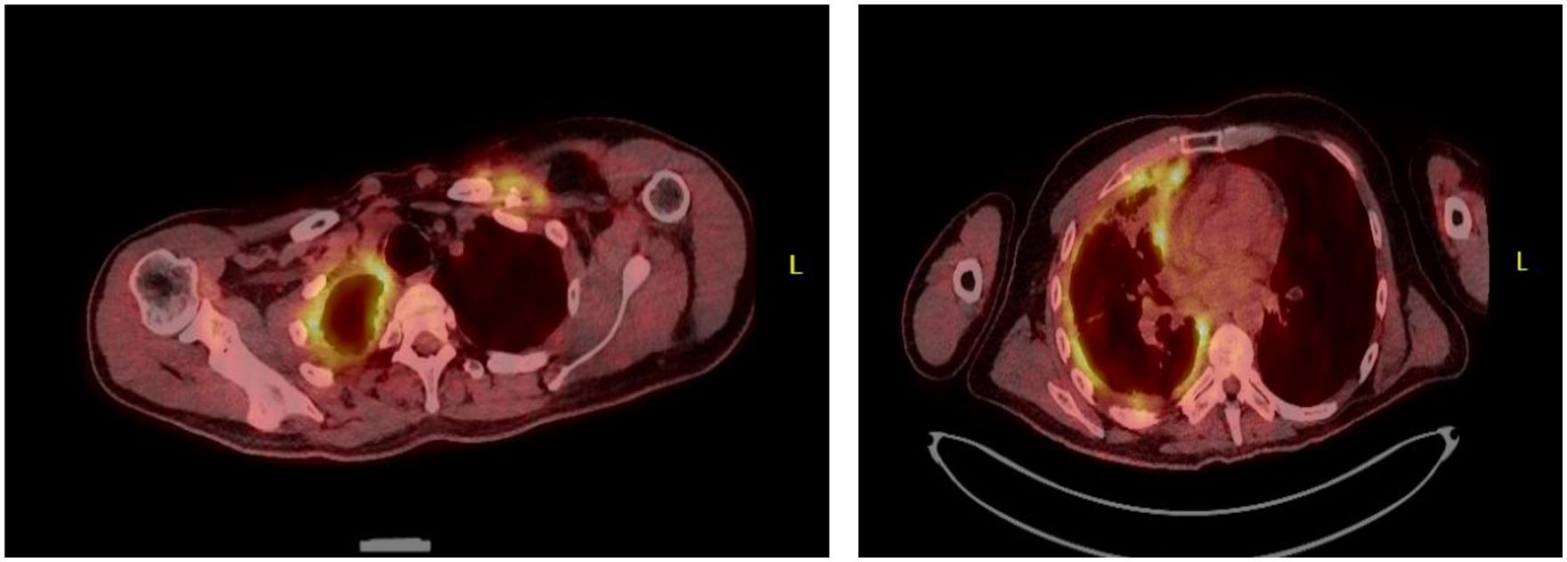
PET-CT Images Showing (A) an FDG-Avid Left Supraclavicular Mass (Arrow) and (B) Ring-Like FDG-Avid Right Pleural Thickening (Arrow) with Associated Atelectasis, Consistent with Metastatic Epithelioid Hemangioendothelioma

Following review of the pathology, a PET scan was recommended in conjunction with an international opinion. The PET scan, as can be seen in the attached image (Figure 1), reported an FDG avid (SUV Max 5) mass in the left supraclavicular region intimately related to the left clavicle, ring-like FDG avid (SUV Max 8) pleural thickening in the right hemithorax with associated atelectasis, volume loss, and FDG avid right para-cardiac nodes. Despite previously inconclusive biopsies, appearances remained concerning for malignant process.

An international opinion on biopsies from Professor Jason L Hornick of Harvard Medical School, Massachusetts, concluded that the findings fit well with epithelioid hemangioendothelioma. Soft tissue examples of this tumour type often arise in association with a large vein. The left shoulder lesion shows muscle infiltrated by an epithelioid neoplasm with mild nuclear atypia and eosinophilic cytoplasm, arranged in nests and cords. By immunohistochemistry performed in his laboratory, the tumour cells were positive for ERG, cm1, and CAMTA1, whereas keratins (AE1/AE3 and PAN-K), MUC4, TFE3, desmin, and sloo protein were negative. CAMTAI expression correlates with the pathognomonic WWTRI: CAMTAI fusion. The pleural biopsy showed a small focus of metastatic epithelioid hemangioendothelioma.

Unfortunately, epithelioid hemangioendothelioma involving the pleura is indicative of metastatic disease and often pursues a highly aggressive clinical course.

This patient commenced on a MEK1 and MEK2 inhibitor, Trametinib, as directed by medical oncology with palliative care input for management of pain. Second-line treatment may include an MTOR inhibitor and a VEGF inhibitor pending patient response to initial treatment. Unfortunately, the patient died from EHE 3 months post commencing on treatment.

## DISCUSSION

Epithelioid hemangioendothelioma is an ultra-rare vascular sarcoma with clinical features ranging from a low-grade malignancy to a high-grade sarcoma. To date, there are no active agents licensed specifically for EHE.^2^ The European Society for Medical Oncology (ESMO) formulated a consensus on the management of EHE; however, it is based on a limited amount of weak evidence. The prevalence is estimated to be <1/1,000,000 with a slightly higher presence in women than men, which usually peaks from age 40-50 years. Clinical presentation can vary pending localized disease or metastatic disease. The clinical presentation of EHE can prove to be somewhat challenging to identify. For patients who do become symptomatic, the symptoms often include: pain, a palpable mass, weight loss, or, in some instances, venous obstruction.^3^

Treatment is dependent on the organ affected by EHE. In this case, if the EHE had been localized, surgical resection would have been the treatment of choice. In the event surgical margins were negative, the estimated cure rate would be 70%-80%.^4^ Surgery can be complemented with radiation therapy when R1 excisions occur. There is, unfortunately, limited evidence to evaluate the effectiveness of radiotherapy in EHE; however, it is considered radiosensitive.

European Society for Medical Oncology was unable to formulate a robust guideline for EHE, simply because it has not been evaluated. Patients with metastatic disease are recommended to commence systemic treatment; however, a standard medical approach has not yet been developed. The use of Trametinib in certain aggressive sarcomas is considered an appropriate off-label strategy when a molecular aberration directly activates the MAPK pathway (e.g., *NF1* loss or specific *BRAF* mutations). This approach aligns with the principles of precision medicine, where therapy is selected based on the specific molecular driver of the tumour, irrespective of its histological classification, when standard options are exhausted.^5^

## CONCLUSION

Epithelioid hemangioendothelioma is a rare cause of pleural disease; however, it should be considered when clinical presentations such as this case are observed. Early diagnosis is requisite for optimum patient outcome. Regrettably, this patient passed away 3 months after commencing treatment.

## ETHICAL STATEMENT

Written informed consent was received for this case report. The signed consent form signed by the patient is located in the patient’s medical notes.

## Data Availability

The data supporting the findings of this case report are available within the article. Due to patient privacy and confidentiality, raw clinical data (including imaging studies and pathology reports) are not publicly available. Anonymized data may be shared upon reasonable request to the corresponding author, subject to institutional and ethical approval.
